# Supplementary material for: Mycobacteriophage Alexphander Gene 94 Encodes an Essential dsDNA-Binding Protein during Lytic Infection
Source: Int J Mol Sci. 2024 Jul 7;25(13):7466. doi: 10.3390/ijms25137466 (PMC11242194; doi:10.3390/ijms25137466)
Supplement: Supplementary file 1 [file ijms-25-07466-s001.zip › ijms-3062505-supplementary.pdf]

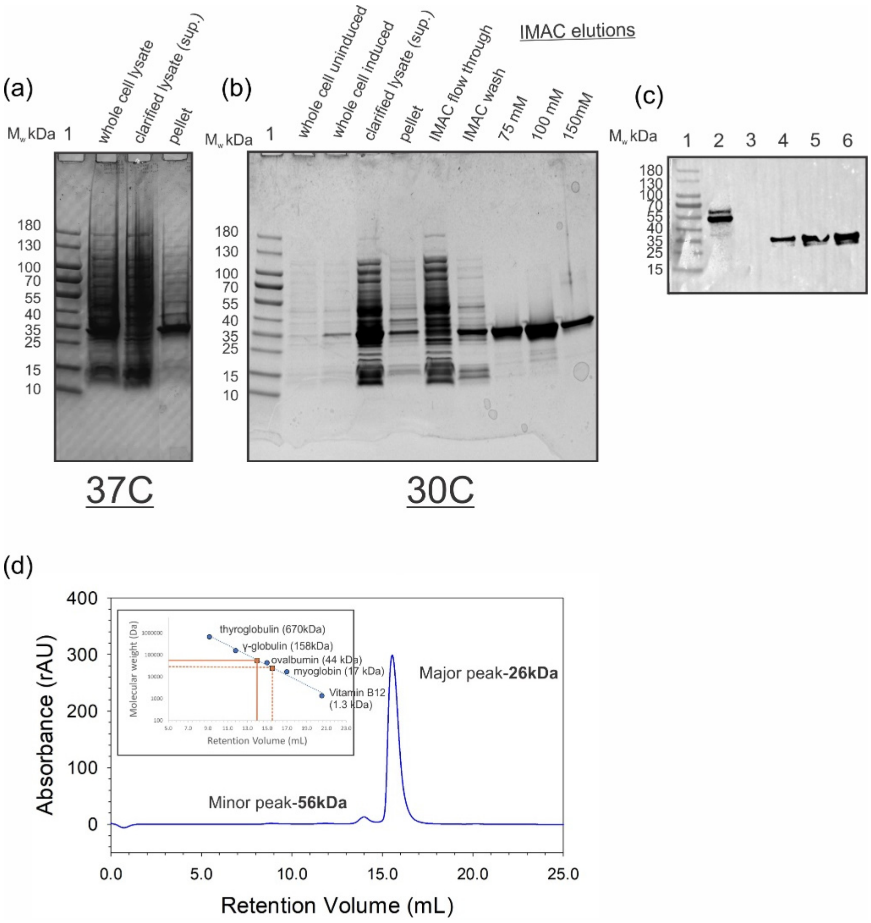

Supplementary Figure S1. gp94 is expressed and remains soluble when induced at 30°C, can form disulfide bonds, and remains monomeric under native conditions. (a) SDS-PAGE showing overexpression of gp94 in whole cell extracts, clarified lysate, and pellet fractions for cells induced at 37°C (lanes 2-4). (b) SDS page gel showing gp94 purity in various *E. coli* fractions during Ni^{2+} affinity purification from Nico21 cells induced at 30°C. lanes 1-10 contain molecular weight ladder, whole cell uninduced, whole cell induced, clarified lysate, pellet, flow through, wash, 50mM elution, 100mM elution, 150mM Elution. (c) Western blot of purified gp94. Lanes 1 and 2 contain molecular weight markers and an E. coli lysate containing control His-tagged protein (HLA), respectively. Lanes 3–6 contain 100 ng, 250 ng, 500 ng and 1 μg of purified gp94, respectively. (d) gel filtration chromatogram for Ni^{2+} affinity purified gp94. A280 is plotted as a function of retention volume. Inset shows a plot of standard retention volume that was used to calculate the molecular weight of gp94 under native conditions. The molecular weights corresponding to retention volume is indicated suggesting that gp94 exists primarily as a monomer under native conditions.
